# A Novel Model for Predicting Associations between Diseases and LncRNA-miRNA Pairs Based on a Newly Constructed Bipartite Network

**DOI:** 10.1155/2018/6789089

**Published:** 2018-05-06

**Authors:** Shunxian Zhou, Zhanwei Xuan, Lei Wang, Pengyao Ping, Tingrui Pei

**Affiliations:** ^1^College of Software and Communication Engineering, Xiangnan University, Chenzhou 423000, China; ^2^College of Information Engineering, Xiangtan University, Xiangtan 411105, China

## Abstract

**Motivation:**

Increasing studies have demonstrated that many human complex diseases are associated with not only microRNAs, but also long-noncoding RNAs (lncRNAs). LncRNAs and microRNA play significant roles in various biological processes. Therefore, developing effective computational models for predicting novel associations between diseases and lncRNA-miRNA pairs (LMPairs) will be beneficial to not only the understanding of disease mechanisms at lncRNA-miRNA level and the detection of disease biomarkers for disease diagnosis, treatment, prognosis, and prevention, but also the understanding of interactions between diseases and LMPairs at disease level.

**Results:**

It is well known that genes with similar functions are often associated with similar diseases. In this article, a novel model named PADLMP for predicting associations between diseases and LMPairs is proposed. In this model, a Disease-LncRNA-miRNA (DLM) tripartite network was designed firstly by integrating the lncRNA-disease association network and miRNA-disease association network; then we constructed the disease-LMPairs bipartite association network based on the DLM network and lncRNA-miRNA association network; finally, we predicted potential associations between diseases and LMPairs based on the newly constructed disease-LMPair network. Simulation results show that PADLMP can achieve AUCs of 0.9318, 0.9090 ± 0.0264, and 0.8950 ± 0.0027 in the LOOCV, 2-fold, and 5-fold cross validation framework, respectively, which demonstrate the reliable prediction performance of PADLMP.

## 1. Introduction

MicroRNAs (miRNAs) are endogenous small and nonencoding RNA molecules, which can regulate gene expression at the posttranscriptional level by combining the 3′ untranslated regions (UTRs) of target mRNAs (UTR) and lead the translation inhibited cleavage of the target mRNAs [1]. Moreover, long-noncoding RNAs (lncRNAs), as the biggest class of noncoding RNAs with length greater than 200 nt, can also regulate gene expression at different levels including transcriptional, posttranscriptional, and epigenetic regulation. Recently, increasing studies demonstrate that lncRNAs and miRNAs play a signification role in the cell proliferation and cell differentiation [2–5] and that the interactions between lncRNAs and microRNAs may have consequences for diseases, explain disease processes, and present opportunities for new therapies [6]. For example, Dey et al. proved that lncRNA H19 would give rise to microRNAs miR-675-3p and miR-675-5p to promote skeletal muscle differentiation and regeneration [7]. Yao et al. discovered that knockdown of lncRNA XIST could exert tumor-suppressive functions in human glioblastoma stem cells by upregulating miR-152 [8]. Wang et al. demonstrated that silencing of lncRNA MALAT1 by miR-101 and miR-217 would inhibit proliferation, migration, and invasion of esophageal squamous cell carcinoma cells [9]. Zhang et al. presented that lncRNA ANRIL indicated a poor prognosis of gastric cancer and promoted tumor growth by epigenetically silencing of miR-99a/miR-449a [10]. You et al. found that miR-449a inhibited cell growth in lung cancer and regulated lncRNA NEAT1 [11]. Emmrich et al. discovered that lncRNAs MONC and MIR100HG would act as oncogenes in AMKL blasts [12]. Leung et al. found that miR-222 and miR-221 upregulated by Ang II were transcribed from a large transcript and knockdown of Lnc-Ang362 would decrease expression of miR-221 and miR-222 and reduce cell proliferation [13]. Zhu et al. discovered that lncRNA H19 and H19-derived miRNA-675 were significantly downregulated in the metastatic prostate cancer cell line M12 compared with the non-meta-static prostate epithelial cell line [14]. Hirata et al. found that lncRNA MALAT1 was associated with miR-205 and promoted aggressive renal cell carcinoma [15]. Zhao and Ren demonstrated that TUG1 knockdown was significantly associated with decreased cell proliferation and promoted apoptosis of breast cancer cells through the regulation of miR-9 [16].

More and more researches have indicated that lncRNA-miRNA interactions are associated with the development of complex diseases, but until now, as far as we know, no prediction models have been proposed for large-scale forecasting of the associations between diseases and LMPairs. However, some prediction models have been reported to infer the associations between diseases and miRNA-miRNA pairs [17–21]. Moreover, there are researches showing that miRNA-miRNA pairs can work cooperatively to regulate an individual gene or cohort of genes that participate in similar processes [18, 22]. Inspired by these existing state-of-the-art methods and ideas for large-scale prediction of the associations between diseases and miRNA-miRNA pairs and based on the reasonable assumption that functionally similar LMPairs tend to be associated with similar diseases, in this paper, a new model named PADLMP is proposed to predict potential associations between diseases and LMPairs. To date, it is the first computational model used to predict disease-LMPairs associations. PADLMP can predict novel disease-LMPairs associations in a large scale by combining the known lncRNA-disease, miRNA-disease, and lncRNA-miRNA associations. To evaluate the prediction performance of the proposed model, evaluation frameworks of leave-one-out cross validation (LOOCV), 2-fold, and 5-fold cross validation were adopted based on the known disease-LMPairs. A series of comparison experiments were also implemented to evaluate the influence of the number of walks on prediction performance. As a result, PADLMP achieved its best performance when the number of walks was set as 2. Specifically, PADLMP achieved value of AUCs of 0.9318, 0.9090 ± 0.0264, and 0.8950 ± 0.0027 in the LOOCV, 2-fold, and 5-fold cross validation framework, respectively. The results of the prediction show that the PADLMP model is feasible and effective in predicting broad-scale disease-LMPairs associations by considering the topology information of the known disease-LMPairs dichotomous network.

## 2. Materials

### 2.1. LncRNA-Disease Associations

Known lncRNA-disease associations were downloaded from different databases such as the lncRNA-disease database lncRNADisease [23], MNDR [24], and Lnc2Cancer [25], respectively, and then, after preprocessing (getting rid of duplicate associations), 2048 distinct experimentally confirmed lncRNA-disease associations that including 1126 lncRNAs and 356 diseases were finally obtained (see Supplementary Table 1). Then we further constructed an adjacency matrix A1 of size 1126 × 356 as the information source.

### 2.2. miRNA-Disease Associations

We also downloaded known disease-miRNA associations from three different databases such as the miR2Disease [26], HMDD [27], and miRCancer [28], respectively. And then, after preprocessing (getting rid of duplicate associations) and mapping these newly obtained miRNAs and diseases to databases of miRBase v21 [29] and Disease Ontology (DO) [30] separately, we finally obtained 4041 disease-miRNA associations including 438 miRNAs and 263 diseases from HMDD, 1839 disease-miRNA associations including 83 cancers and 327 miRNAs from miRCancer, and 1487 disease-miRNA associations including 107 diseases and 276 miRNAs from miR2Disease (see Supplementary Table 2).

### 2.3. LncRNA-miRNA Associations

In this section, we downloaded two versions (2015 Version and 2017 Version) of lncRNA-miRNA association datasets from the starBasev2.0 database [31], which provided the most comprehensive experimentally confirmed lncRNA-miRNA interactions based on large-scale CLIP-Seq data. And then, after preprocessing (including elimination of duplicate values, erroneous data, and disorganized data), 20324 lncRNA-miRNA interactions including 494 miRNAs and 1127 lncRNAs were obtained finally (see Supplementary Table 3).

## 3. Methods

### 3.1. Methods Overview

**Figure 1:**
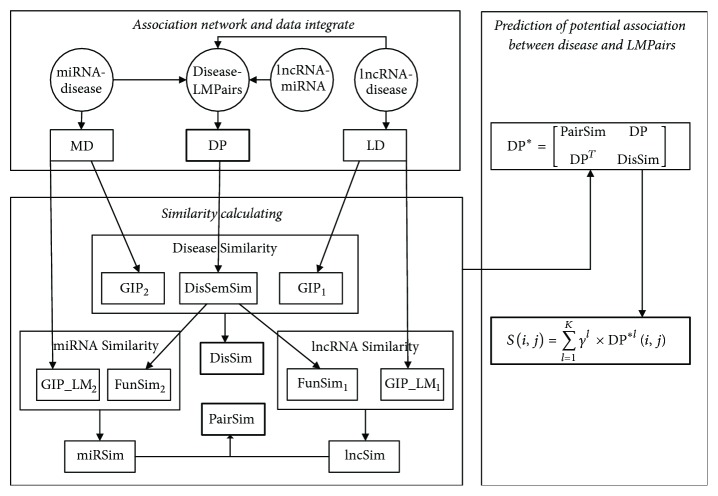
Flowchart of PADLMP based on known miRNA-disease, lncRNA-disease, and lncRNA-miRNA association network.

In order to predict potential novel associations between diseases and LMPairs, a new model named PADLMP is proposed, which consists of three steps (Figure 1). First, the construction of association network and data integrate. Second, the similarities for lncRNAs, diseases, miRNAs, and lncRNA-miRNA pairs are calculated based on the association network. Finally, potential associations between disease and LMPairs are inferred.

### 3.2. Construct the Associated Network

#### 3.2.1. LncRNA-Disease Network, Disease-miRNA Network, and LncRNA-miRNA Network

Based on these newly obtained known lncRNA-disease associations, we constructed the lncRNA-disease bipartite network *G*_1_ = (*V*_1_, *E*_1_) according to the following steps.

**Step 1 .**

Let *V*_*l*1_ be the set of newly collected 1126 lncRNAs, let *V*_*d*1_ be the set of newly collected 356 diseases, and *V*_1_ = *V*_*l*1_ ∪ *V*_*d*1_, then we can obtain the vertex set *V*_1_ of *G*_1_.

**Step 2 .**

∀*l*_*i*_ ∈ *V*_*l*1_, if there is *d*_*j*_ ∈ *V*_*d*1_ satisfying the fact that the association between *l*_*i*_ and *d*_*j*_ belongs to the set of newly collected 2048 lncRNA-disease associations, then we define that there is an edge between *l*_*i*_ and *d*_*j*_ in *G*_1_, and by this way, we can obtain the edge set *E*_1_ of *G*_1_. Obviously, *E*_1_ is composed of these newly collected 2048 lncRNA-disease associations.

Similar to *G*_1_, we constructed the disease-miRNA bipartite network *G*_2_ = (*V*_2_, *E*_2_) according to the following steps.

**Step 1 .**

Let *V*_*m*1_ be the set of all these newly collected miRNAs, let *V*_*d*2_ be the set of all these newly collected diseases, and *V*_2_ = *V*_*m*1_ ∪ *V*_*d*2_, then we can obtain the vertex set *V*_2_ of *G*_2_.

**Step 2 .**

∀*m*_*i*_ ∈ *V*_*m*1_, if there is *d*_*j*_ ∈ *V*_*d*2_ satisfying the fact that the association between *m*_*i*_ and *d*_*j*_ belongs to the set of all these newly collected disease-miRNA associations, then we define that there is an edge between *m*_*i*_ and *d*_*j*_ in *G*_2_, and by this way, we can obtain the edge set *E*_2_ of *G*_2_. Obviously, *E*_2_ is composed of all these newly collected disease-miRNA associations.

We also constructed the lncRNA-miRNA bipartite network *G*_3_ = (*V*_3_, *E*_3_) according to the following steps.

**Step 1 .**

Let *V*_*l*2_ be the set of newly collected 1127 lncRNAs, let *V*_*m*2_ be the set of newly collected 494 miRNAs, and *V*_3_ = *V*_*m*2_ ∪ *V*_*l*2_, then we can obtain the vertex set *V*_3_ of *G*_3_.

**Step 2 .**

∀*l*_*i*_ ∈ *V*_*l*2_, if there is *m*_*j*_ ∈ *V*_*m*2_ satisfying the fact that the association between *l*_*i*_ and *m*_*j*_ belongs to the set of newly collected 18286 lncRNA-miRNA associations, then we define that there is an edge between *l*_*i*_ and *m*_*j*_ in *G*_3_, and by this way, we can obtain the edge set *E*_3_ of *G*_3_. Obviously, *E*_3_ is composed of these newly collected 20324 lncRNA-miRNA associations.

#### 3.2.2. Disease-LncRNA-miRNA Network

Based on above newly constructed bipartite networks such as *G*_1_, *G*_2_, and *G*_3_, we constructed a new tripartite network *G*_4_ = (*V*_4_, *E*_4_) according to the following steps.

**Step 1 .**

Let *V*_*l*′_ = *V*_*l*1_∩*V*_*l*2_, *V*_*m*′_ = *V*_*m*1_∩*V*_*m*2_, and *V*_*d*3_ = *V*_*d*1_∩*V*_*d*2_. ∀*d*_*i*_ ∈ *V*_*d*3_, if there are *l*_*j*_ ∈ *V*_*l*′_ and *m*_*k*_ ∈ *V*_*m*′_ satisfying the fact that the association between *d*_*i*_ and *l*_*j*_ belongs to *E*_1_, the association between *d*_*i*_ and *m*_*k*_ belongs to *E*_2_, and the association between *l*_*j*_ and *m*_*k*_ belongs to *E*_3_ simultaneously. Then we define that there are an edge between *d*_*i*_ and *l*_*j*_, an edge between *d*_*i*_ and *m*_*k*_, and an edge between *l*_*j*_ and *m*_*k*_ in *G*_4_ separately, and by this way, we can obtain the edge set *E*_4_ of *G*_4_.

**Step 2 .**

Let *V*_*l*_⊆*V*_*l*′_ satisfying the fact that ∀*l*_*i*_ ∈ *V*_*l*_ there is *d*_*j*_ ∈ *V*_*d*3_ satisfying the fact that the association between *d*_*j*_ and *l*_*i*_ belongs to *E*_4_. Let *V*_*m*_⊆*V*_*m*′_ satisfying the fact that ∀*m*_*i*_ ∈ *V*_*m*_ there is *d*_*j*_ ∈ *V*_*d*3_ satisfying that the association between *d*_*j*_ and *m*_*i*_ belongs to *E*_4_. Let *V*_4_ = *V*_*l*_ ∪ *V*_*m*_ ∪ *V*_*d*3_, then we can obtain the vertex set *V*_4_ of *G*_4_.

#### 3.2.3. Disease-LMPairs Network

Based on above newly obtained tripartite Disease-LncRNA-miRNA network *G*_4_, we constructed a new bipartite disease-LMPairs network *G* = (*V*, *E*) according to the following steps.

**Step 1 .**

∀*l*_*i*_ ∈ *V*_*l*_ and *m*_*j*_ ∈ *V*_*m*_, let *p*_*ij*_ = (*l*_*i*_, *m*_*j*_) and *V*_*p*_ = {*p*_*ij*_} where *i* ∈ [1, |*V*_*l*_|] and *j* ∈ [1, |*V*_*m*_|], then we define *V* = *V*_*d*3_ ∪ *V*_*p*_, and by this way, we can obtain the vertex set *V* of *G*.

**Step 2 .**

∀*d*_*k*_ ∈ *V*_*d*3_, there is *p*_*ij*_ = (*l*_*i*_, *m*_*j*_) ∈ *V*_*p*_ satisfying the fact that the association between *d*_*k*_ and *l*_*i*_ belongs to *E*_1_, the association between *d*_*k*_ and *m*_*j*_ belongs to *E*_2_, and the association between *l*_*i*_ and *m*_*j*_ belongs to *E*_3_ simultaneously. Then we define that there is an edge between *d*_*k*_ and *p*_*ij*_ in *G*, and by this way, we can obtain the edge set *E* of *G*.

To make it easier to understand the construction of the network, we list in “The Meaning of Vertex and Edges in the Networks” each of the vertices, edges, and their meanings that appear in Sections 3.2.1, 3.2.2, and 3.2.3.

### 3.3. Calculation the Similarity of Disease

#### 3.3.1. Calculation of the Disease Semantic Similarity (DisSemSim)

Firstly, we downloaded* MeSH* descriptors from the National Library of Medicine and curated the names of diseases using the standard* MeSH *disease terms. Next, we represented the relationship of different diseases by a structure of directed acyclic graph (DAG) such as DAG(*D*) = (*T*(*D*), *E*(*D*)). Here, *T*(*D*) represented the node set including node *D* and its ancestor nodes, and *E*(*D*) denoted the edge set of corresponding direct links from a parent node to a child node, which represented the relationship between different diseases [32]. Then, based on the disease DAG, the contribution of an ancestor node *d* to the semantic value of disease *D* and the contribution of the semantic value of disease *D* itself can be calculated by the following two equations, respectively:

(1) DDd=1if  d=D

(2) DVD=∑d∈TDDDd,

where *D*_*D*_(*d*) represents the contribution of an ancestor node *d* to the semantic value of disease *D*, DV(*D*) represents the contribution of the semantic value of disease *D* itself, and Δ is the semantic contribution decay factor with value between 0 and 1. The function of parameter Δ is to guarantee that, as the distances between disease *D* and its ancestor disease *d* increase, the contribution of *d* to *D* will progressively decrease. Moreover, from the above formula (1), it is easy to see that it is also reasonable to define the contribution of *D* to itself as 1. In addition, according to the experimental results of some previous state-of-the-art methods [33, 34], we will set the value of Δ as 0.5 in this paper.

In order to measure disease semantic similarity that two diseases with more common ancestor nodes in the DAG shall have higher semantic similarity, based on the assumption, we can define the semantic similarity between two diseases *d*_*i*_ and *d*_*j*_ as follows:

(3) DisSemSimdi,dj=∑t∈Tdi∩TdjDdit+DdjtDVdi+DVdj,

where *T*(*d*_*i*_) and *T*(*d*_*j*_) represented the node sets of the DAG of *d*_*i*_ and *d*_*j*_, respectively.

#### 3.3.2. Calculation of the Gaussian Interaction Profile Kernel Similarity for Diseases (*GIPSim*)

According to the assumption that functionally similar genes tend to be associated with similar diseases, we can integrate the topologic information of known miRNA-disease association network and lncRNA-disease association network to measure the disease similarity. Moreover, in this section, we will adopt Gaussian Interaction Profile Kernel to calculate the similarity of diseases. Firstly, based on the networks such as *G*_1_ and *G*_2_ constructed above, we can obtain two adjacency matrices such as *Y*_1_ (or *Y*_2_) as follows. For any given lncRNA *l*_*i*_ (or miRNA *m*_*i*_) and disease *d*_*j*_, while *k* takes 1 or 2, we define that

(4) $$\begin{array}{c} Y_{k}\left(\;i,j\;\right)=\left\{\begin{array}{cc} 1 & \text{exist an edge between }l_{i}\left(m_{i}\right)\text{ and disease }\;d_{j}\text{ in }G_{1}\left(G_{2}\right)\\ 0 & \text{otherwise.} \end{array}\right. \end{array}$$

Hence, let IP_*k*_(*d*_*i*_) denote the *i*th column of matrix *Y*_*k*_, then we can calculate the Gaussian Kernel Similarity between the diseases *d*_*i*_ and *d*_*j*_ based on their interaction profiles as follows:

(5) GIPkdi,dj=exp⁡−γkIPkdi−IPkdj2γk=11/nk∑i=1nkIPkdi2,

where the parameter *n*_*k*_ denotes the number of diseases in *G*_*k*_  (*k* = 1,2).

Based on formula (5), we can adopt squared root approach to calculate the Gaussian Interaction Profile Kernel Similarity for diseases as follows:

(6) $$\begin{array}{c} GIPSim\left(\;d_{i},d_{j}\;\right)={\left(\;{GIP}_{1}\left(\;d_{i},d_{j}\;\right)\times {GIP}_{2}\left(\;d_{i},d_{j}\;\right)\;\right)}^{1/2}. \end{array}$$

#### 3.3.3. Calculation of the Integrated Similarity between Disease

Based on these formulas presented above, we can finally define the similarity measurement between diseases *d*_*i*_ and *d*_*j*_ as follows:

(7) $$\begin{array}{c} \text{DisSim}\left(\;d_{i},d_{j}\;\right)=\left\{\begin{array}{cc} \text{GIPSim}\left(d_{i},d_{j}\right) & \text{if DisSemSim}\left(d_{i},d_{j}\right)=0\\ \frac{\text{GIPSim}\left(d_{i},d_{j}\right)+\text{DisSemSim}\left(d_{i},d_{j}\right)}{2} & \text{otherwise.} \end{array}\right. \end{array}$$

### 3.4. Calculation of the Similarity between LncRNAs (miRNAs)

#### 3.4.1. Calculation of the LncRNA (miRNA) Functional Similarity

For any given two lncRNAs (miRNAs) such as *l*_*i*_(*m*_*i*_) and *l*_*j*_(*m*_*j*_), let *DT*_1_ = {*dt*_11_, *dt*_12_,…, *dt*_1*m*_} be all diseases related to *l*_*i*_(*m*_*i*_) in *G*_1_(*G*_2_) and let *DT*_2_ = {*dt*_21_, *dt*_22_,…, *dt*_2*n*_} be all diseases related to *l*_*j*_(*m*_*j*_) in *G*_1_(*G*_2_), then we can define the functional similarity between *l*_*i*_(*m*_*i*_) and *l*_*j*_(*m*_*j*_) as follows (*k* = 1, *v* = *l* or *k* = 2, *v* = *m*):

(8) FunSimkvi,vj=∑1≤p≤mSemSimsdt1p,DT2+∑1≤p≤nSemSimsdt2p,DT1m+n,

where

(9) SemSimsdt1p,DT2=max1≤l≤n⁡DisSemSimdt1p,dt2l.

#### 3.4.2. Calculation of the Gaussian Interaction Profile Kernel Similarity for IncRNAs (miRNA)

For any given two lncRNAs (miRNAs) such as *l*_*i*_(*m*_*i*_) and *l*_*j*_(*m*_*j*_), in a similar way to the calculation of GIP_1_, GIP_2_ can be obtained as follows (*k* = 1, *v* = *l* or *k* = 2, *v* = *m*):

(10) GIP_LMkvi,vj=exp⁡−γkIPkvi−IPkvj2γk=11/nk∑i=1nkIPkvi2,

where IP_*k*_(*v*_*i*_) and IP_*k*_(*v*_*j*_) are the *i*th row and the *j*th row in matrix *Y*_*k*_, respectively, and *n*_*k*_ is the number of lncRNAs (miRNA) in *G*_*k*_.

#### 3.4.3. Calculation of the Integrated Similarity between IncRNAs (miRNAs)

Based on these formulas presented above, we can finally define the similarity measurement between lncRNAs *l*_*i*_ and *l*_*j*_ as follows:

(11) lncSimli,lj=FunSim1li,lj+GIP_LM1li,lj2miRSimmi,mj=FunSim2mi,mj+GIP_LM2mi,mj2.

### 3.5. Similarity for LncRNA-miRNA Pairs (LMPairSim)

Based on the bipartite disease-LMPairs network *G* constructed above, for any given two lncRNA-miRNA pairs *p*_*ij*_ = (*l*_*i*_, *m*_*j*_) and *p*_*ab*_ = (*l*_*a*_, *m*_*b*_), we can calculate the similarity between them according to the following three different ways:

*(1) Average Approach*

(12) LMPairSimPij,Pab=lncSimli,la+miRSimmj,mb2.

*(2) Squared Root Approach*

(13) LMPairSimPij,Pab=lncSimli,la×miRSimmj,mb1/2.

*(3) Centre Distance Approach*

(14) $$\begin{array}{c} LMPairSim\left(\;d_{i},d_{j}\;\right)=\sqrt{{{\left(lncSim\left(l_{i},l_{a}\right)-\text{AvglncSim}\right)}^{2}+\left(miRSim\left(m_{j},m_{b}\right)-\text{AvgmiRSim}\right)}^{2}}, \end{array}$$

where

(15) AvglncSim=∑i=1nl∑j=1nllncSimli,ljnl2,AvgmiRSim=∑i=1nm∑j=1nmmiRSimmj,minm2.

### 3.6. Prediction of Potential Associations between Diseases and LMPairs

Inspired by the KATZ method in social networks [35], disease-gene correlation prediction [36], and lncRNA-association prediction of disease [37], we explored the PADLMP measure by developing a new computational model for predicting disease-LMPairs associations (see Figure 1). Obviously, based on the formulas (12), (13), (14), and (15), let *N*_*d*_ denote the number of diseases in *G*, *N*_*p*_ denote the number of LMPairs in *G*, *N*_*l*_ denote the number of lncRNAs in *G*, and *N*_*m*_ denote the number of miRNAs in *G*, respectively, then we can obtain a *N*_*d*_ × *N*_*d*_ dimensional matrix DisSim and *N*_*p*_ × *N*_*p*_ dimensional matrix PairSim. Moreover, we can construct *N*_*p*_ × *N*_*p*_ dimensional adjacency matrices DP as follows:

(16) DPi,j=1exist  an  edge  between  di  and  pj  in  G0otherwise,

where *d*_*i*_ denotes the *i*th disease in *G* and *p*_*j*_ denotes the *j*th LMPair in *G*

Hence, inspired by the approach based on KATZHMDA [38] and KATZ [35], we can construct an integrated matrix DP^*∗*^ for further predicting the potential associations between diseases and LMPairs as follows:

(17) $$\begin{array}{c} {DP}^{*}=\left[\begin{array}{cc} PairSim & DP\\ {DP}^{T} & DisSim \end{array}\right]. \end{array}$$

Based on the integrated matrix DP^*∗*^ constructed above and letting *V*_*p*_ = {*P*_1_, *P*_2_,…, *P*_*Np*_}, then, for any given lncRNA-miRNA pair *p*_*i*_ ∈ *V*_*p*_ and diseases node *d*_*j*_ ∈ *V*_*d*_, the probability of potential association between *p*_*i*_ and *d*_*k*_ can be obtained as follows:

(18) $$\begin{array}{c} S\left(\;i,j\;\right)=\overset{K}{\underset{l=1}{\sum }}\gamma^{l}\times {{DP}^{*}}^{l}\left(\;i,j\;\right), \end{array}$$

where the parameter *K* is an integer bigger than 1 and the parameter *γ* satisfies 0 < *γ* < 1.

Additionally, according to the above formula (18), it is obvious that the (*N*_*p*_ + *N*_*d*_)×(*N*_*p*_ + *N*_*d*_) dimensional matrix *S* depicts the possibilities of all associations between diseases and LMPairs in *G*, and it can be further modified into the following form:

(19) $$\begin{array}{c} S=\underset{l\geq 1}{\sum }\gamma^{l}\times {{DP}^{*}}^{l}={\left(\;I-\gamma \times {DP}^{*}\;\right)}^{-1}-I=\left[\begin{array}{cc} S_{11} & S_{12}\\ S_{21} & S_{22} \end{array}\right], \end{array}$$

where *S*_11_ is *N*_*p*_ × *N*_*p*_ dimensional matrix, *S*_12_ is *N*_*p*_ × *N*_*d*_ dimensional matrix, *S*_21_ is *N*_*d*_ × *N*_*p*_ dimensional matrix, and *S*_22_ is *N*_*d*_ × *N*_*d*_ dimensional matrix.

From formula (19), it is easily to know that *S*_12_ is exactly the* final prediction result matrix*, which includes all of the potential associations between diseases and LMPairs in *G*. In addition, considering that a long walker in a sparse network may be less meaningful, it will disrupt association prediction, so we set *K* to 2, 3, and 4 here. Then, final prediction result matrix could be represented by matrix DP, PairSim, and DisSim based on aforementioned equation (19).

While *K* = 2, there is
  (20) S122=γ×DP+γ2×PairSim×DP+DP×DisSim.
While *K* = 3, there is
  (21) S123=S122+γ3×DP×DPT×DP+PairSim2×DP+PairSim×DP×DisSim+DP×DisSim2.
While *K* = 4, there is
  (22) S124=S123+γ4×PairSim3×DP+DP×DPT×PairSim×DP+PairSim×DP×DPT×DP+DP×DisSim×DPT×DP+γ4×DP×DPT×DP×DisSim+PairSim2×DP×DisSim+PairSim×DP×DisSim2+DP×DisSim3.

## 4. Results

In order to estimate the prediction performance of our newly proposed model PADLMP, the leave-one-out cross validation (LOOCV) procedure was adopted based on the positive samples of disease-LMPair associations. In the LOOCV validation framework, each known disease-LMPair association is used as a test sample, and the remaining disease-LMPairs association is used as a training sample for model learning. In particular, all the disease-LMPairs without known relevance proofs will be considered as candidate samples. In the LOOCV, we can obtain the rank of each left-out testing sample relative to candidate samples, and if the test samples are with a prediction level higher than a given threshold, then it will be considered to be successfully predicted. The corresponding true positive rates (TPR, sensitivity) and false positive rates (FPR, 1 − specificity) could be obtained by setting different thresholds. Here, sensitivity measures the percentage of test samples which are predicted with a higher rank than given threshold, specificity is calculated as the percentage of negative samples ranked below a given threshold. The receiver operating characteristics (ROC) curves can be drawn by plotting TPR versus FPR by different thresholds. In order to evaluate the predictive performance of PADLMP, the areas under the ROC curve (AUC) were further calculated. 1 of the AUC value showed a perfect prediction, while 0.5 of the AUC value represented purely random performance.

**Figure 2:**
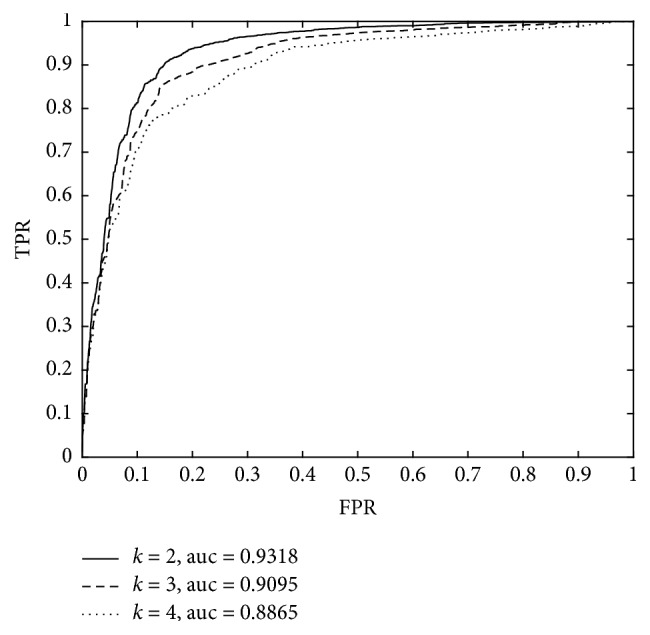
Prediction performance of PADLMP while *K* takes different values in LOOCV.

From the above, we can find that there are some parameters such as *K*, *γ* adopted in our prediction model PADLMP. It is obvious that these parameters are critical to the prediction performance of our model. Moreover, in Section 3.5, three different ways have been proposed to calculate the similarity for lncRNA-miRNA pairs (LMPairSim), then we need to further evaluate the performances of these three different ways also. Hence, in this section, based on the validation framework of LOOCV, we implemented a series of comparison experiments to evaluate the influence of these parameters, and the simulation results were shown in Figure 2. As a result, from Figure 2, it is easy to see that PADLMP can achieve the best prediction performance while *K* was set to 2. Additionally, as for other parameters *γ*, during simulations, we will set *γ* as 0.01 based on the empirical values given by previous state-of-the-art works [37, 39–41]. Moreover, in the LOOCV, for the similarity calculation of LMPairSim, we use formulas (12), (13), and (14) in order and then select the formula that obtains the maximum AUC value. As a result, the AUC value of 0.9318, 0.9262, and 0.9247 were obtained when selecting formulas (12), (14), and (13), respectively.

**Figure 3:**
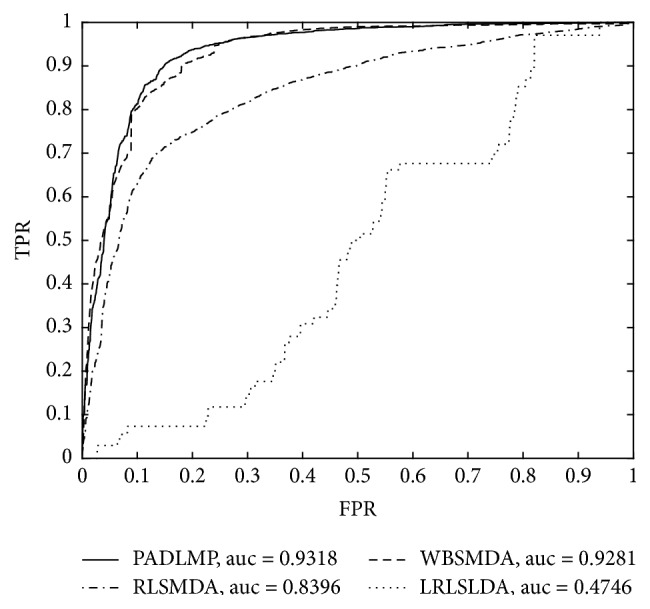
Comparison between PADLMP and RLSMDA, WBSMDA, and LRLSLDA in LOOCV.

Furthermore, we also compared the performance of our prediction model PADLMP with that of the RLSMDA [42], WBSMDA [39], and LRLSLDA [41] in LOOCV, since negative samples were not required in PADLMP, RLSMDA, WBSMDA, and LRLSLDA. The simulation results were shown in Figure 3. It is easy to see that PADLMP can achieve a reliable AUC of 0.9318, which is much higher than the AUC of 0.8104 and 0.9281 achieved by RLSMDA, WBSMDA, LRLSLDA, respectively, In addition, we can clearly see that the AUC value of the model LRSLDA is less than 0.5, which is obviously unreasonable. So based on prior knowledge [43], we subtract this value less than 0.5 from 1 and then we get the AUC value of LRSLDA being 0.5254.

Moreover, in order to further evaluate the prediction performance of PADLMP, the *k*-fold cross validation was also implemented, in which all the known disease-LMPair association samples were randomly equally divided into *k* parts, and *k* − 1 parts were then used as training samples for model learning while the rest part was used as testing samples for model evaluation. Specifically, in this section, considering time complexity and costs, we would only implement 2-fold and 5-fold cross validation to evaluate the prediction performance of PADLMP. In a similar way to that of LOOCV, all the disease-LMPairs without known relevance evidences would be considered as candidate samples in the *k*-fold cross validation. Next, in case of the prediction performance bias caused by random division of the testing samples, we would repeat the random division of the testing samples and our simulations for 100 times, and then, the corresponding ROC curves and AUCs would be obtained in a similar way to that of LOOCV. Simulation results were shown in Table 1, and as a result, from the Table 1, it is easy to see that PADLMP can achieve the best prediction performance with average AUCs of 0.9090 and 0.8950 with Standard Deviation (STD) of 0.0264 and 0.0027 in the 2-fold and 5-fold cross validation, respectively, while setting *K* = 2.

From the above descriptions, it is obvious that the newly proposed model PADLMP can achieve a reliable and effective prediction performance in both LOOCV and *k*-fold cross validation. Therefore, we released the potential disease-LMPair associations with higher predicted relevance scores publicly (see Supplementary Table 4) and anticipated that these disease-LMPair associations may offer valuable information and clues for corresponding biological experiments and would be confirmed by experimental observations in the future.

## 5. Case Studies

Colon cancer is a malignant tumor that is usually found at the borders of rectum and sigmoid colon [44]. This is the third most common cancer and the third leading cause of cancer death in men and women in the United States [45]. However, patients with early colon tumors only suffer from subtle symptoms [46], which make the disease difficult to be detected. In addition, worse, it is reported that its incidence has an upward trend in recent years [47]. Therefore, there is an urgent need to predict potential miRNAs and lncRNAs associated with colon tumors. With the help of modern medicine, many miRNAs have been shown to be associated with colon tumors. For example, miRNA-145 targets the insulin receptor substrate-1 and thus inhibits the growth of colon cancer cells [48].

Moreover, as the second largest cause of cancer deaths in women, breast cancer accounts for the total number of cancers in women 22% [49, 50]. Breast cancer is caused by a variety of molecular changes, traditionally diagnosed by histopathological features such as tumor size, grading, and lymph node status [49]. Studies have shown that lncRNAs and miRNAs play important role in many biological processes and are closely related to the formation of various cancers, including breast cancer [51, 52]. In order to better diagnose and treat breast cancer, it is necessary to predict breast cancer-related lncRNA or miRNAs and to identify lncRNA and miRNA biomarkers [52].

In addition, prostate cancer is a malignant tumor derived from prostate epithelial cells [53]. There are many factors, including age, family history of disease, and race, which may increase the risk of prostate neoplasms [54]. So far, many miRNAs and lncRNAs, such as miRNA has-let-7a-5p and lncRNA XIST in the prostate, have been found to be associated with prostate tumors.

As described previously, PADLMP has been demonstrated that it can achieve a reliable and effective prediction performance. Hence, in this section, case studies about above three kinds of important cancers based on top 5% of predicted results will be implemented to show the prediction performance of PADLMP. As illustrated in Table 2, the prediction results have been verified based on the recent updates in the databases such as lncRNADisease, MNDR v2.0, starBase v2.0, HMDD, miR2Disease, and miRCancer.

In Table 2, “#” and “*∗*” stand for databases of lncRNA-disease and MNDR v2.0, respectively, which consist of known disease-lncRNA associations. “$” stands for starBase v2.0 database, which consists of known lncRNA-miRNA associations. “!,”“&,” and “+” stand for databases of HMDD, miR2Disease, and miRCancer, respectively, which consist of known disease-miRNA associations.

## 6. Discussion and Conclusion

Accumulating evidences show that the interaction of lncRNA-miRNAs is involved in the formation of many complex human diseases, such as breast cancer [16]; however, to our knowledge, there are no prediction models proposed for large scale forecasting the associations between diseases and LMPairs. Hence, based on the existing miRNA-disease associations, lncRNA-disease associations, lncRNA-miRNA interactions, and the assumption that genes with similar functions are often associated with similar diseases, we proposed a novel prediction model PADLMP to infer potential associations between diseases and LMPairs.

In this paper, we achieved the following contributions mainly: (1) we proposed the first computational model PADLMP for large-scale prediction of disease-LMPair associations, which can predict potential associations between diseases and lncRNA-miRNA pairs effectively. (2) We transformed the tripartite Disease-LncRNA-miRNA network into a bipartite disease-LMPair network, which greatly reduced the complexity of our prediction model. (3) Negative samples were not required in our prediction model.

However, although PADLMP is a powerful tool to infer novel associations between diseases and lncRNA-miRNA pairs, there are some limitations still existing in our method. For example, firstly, although we introduced semantic similarity for diseases and LMPairs, but the calculation of Gaussian Interaction Profile Kernel Similarity greatly relied on known disease-lncRNA associations, disease-miRNA associations, and disease-LMPairs associations. Therefore, it would cause inevitable bias towards those well-investigated diseases and LMPairs. Secondly, PADLMP could not be applied to unknown diseases and LMPairs, which were poorly investigated and had not any known associations. In the future, we will try to design new methods that do not rely on the topological information of disease-LMPair association network to solve these limitations.

## Figures and Tables

**Table 1:** Prediction performance of PADLMP while *K* was set to different values in the 2-fold and 5-fold cross validation, respectively.

5-fold	*K* = 2	*K* = 3	*K* = 4
AUC	0.8950	0.8367	0.7724
STD	0.0027	0.0050	0.0109

2-fold	*K* = 2	*K* = 3	*K* = 4

AUC	0.9090	0.8709	0.8518
STD	0.0264	0.0361	0.0441

**Table 2:** PADLMP was applied to three kinds of important cancer.

Disease	LncRNA	miRNA	Evidence
Colon cancer	MALAT1	hsa-miR-145-5p	#, $, !
Colon cancer	MALAT1	hsa-miR-181a-5p	#, $, +
Colon cancer	MALAT1	hsa-miR-155-5p	#, $, !
Colon cancer	MALAT1	hsa-miR-101-3p	#, $, !
Colon cancer	MALAT1	hsa-miR-25-3p	#, $, +
Colon cancer	MALAT1	hsa-miR-143-3p	#, $, !
Colon cancer	MALAT1	hsa-miR-200c-3p	#, $, !
Colon cancer	MALAT1	hsa-miR-429	#, $, +
Colon cancer	MALAT1	hsa-miR-22-3p	#, $, !
Colon cancer	MALAT1	hsa-miR-320a	#, $, +
Breast cancer	XIST	hsa-let-7b-5p	#, $, !
Breast cancer	XIST	hsa-let-7a-5p	#, $, !
Breast cancer	XIST	hsa-miR-146a-5p	#, $, !
Breast cancer	XIST	hsa-miR-27a-3p	#, $, !
Breast cancer	XIST	hsa-let-7c-5p	#, $, !
Breast cancer	XIST	hsa-miR-181b-5p	#, $, !
Breast cancer	XIST	hsa-miR-181a-5p	#, $, !
Breast cancer	XIST	hsa-miR-34a-5p	#, $, !
Breast cancer	XIST	hsa-miR-25-3p	#, $, !
Breast cancer	XIST	hsa-miR-30a-5p	#, $, !
Prostate cancer	XIST	hsa-let-7b-5p	#, $, &
Prostate cancer	XIST	hsa-miR-146a-5p	*∗*, $, &
Prostate cancer	XIST	hsa-miR-27a-3p	*∗*, $, &
Prostate cancer	XIST	hsa-miR-7a-5p	*∗*, $, &
Prostate cancer	XIST	hsa-miR-30a-5p	*∗*, $, &
Prostate cancer	XIST	hsa-miR-34a-5p	*∗*, $, &
Prostate cancer	XIST	hsa-miR-155-5p	*∗*, $, +
Prostate cancer	XIST	hsa-miR-124-3p	*∗*, $, +
Prostate cancer	XIST	hsa-miR-181b-5p	*∗*, $, &
Prostate cancer	XIST	hsa-miR-25-3p	*∗*, $, &

## The Meaning of Vertex and Edges in the Networks

*G*_1_: LncRNA-disease bipartite network
*G*_2_: Disease-miRNA bipartite network
*G*_3_: LncRNA-miRNA bipartite network
*G*_4_: Disease-LncRNA-miRNA tripartite network
*G*: Disease-LMPairs bipartite network
*V*_*l*1_: LncRNA in lncRNA-disease association
*V*_*l*2_: LncRNA in lncRNA-miRNA association
*V*_*d*1_: Disease in lncRNA-disease association
*V*_*d*2_: Disease in miRNA-disease association
*V*_*m*1_: miRNA in miRNA-disease association
*V*_*m*2_: Disease in lncRNA-miRNA association
*V*_*l*′_: *V*_*l*′_ = *V*_*l*1_∩*V*_*l*2_
*V*_*m*′_: *V*_*m*′_ = *V*_*m*1_∩*V*_*m*2_
*V*_*d*3_: *V*_*d*3_ = *V*_*d*1_∩*V*_*d*2_
*V*_*l*_: *V*_*l*_⊆*V*_*l*′_
*V*_*m*_: *V*_*m*_⊆*V*_*m*′_
*p*_*ij*_: LncRNA-miRNA pair (*l*_*i*_, *m*_*j*_)
*E*_1_: Edge of *G*_1_, lncRNA *l*_*i*_ associated with disease *d*_*j*_ if edge 〈*l*_*i*_, *d*_*j*_〉 ∈ *E*_1_
*E*_2_: Edge of *G*_2_, miRNA *m*_*i*_ associated with disease *d*_*j*_ if edge 〈*m*_*i*_, *d*_*j*_〉 ∈ *E*_2_
*E*_3_: Edge of *G*_3_, lncRNA *l*_*i*_ associated with miRNA *m*_*j*_ if edge 〈*l*_*i*_, *m*_*j*_〉 ∈ *E*_3_
*E*_4_: Edge of *G*_4_, lncRNA *l*_*i*_ associated with disease *d*_*j*_ if edge 〈*l*_*i*_, *d*_*j*_〉 ∈ *E*_4_ or lncRNA *l*_*i*_ associated with miRNA *m*_*k*_ if edge 〈*l*_*i*_, *m*_*k*_〉 ∈ *E*_4_ or miRNA *m*_*k*_ associated with disease *d*_*j*_ if edge 〈*m*_*k*_, *d*_*j*_〉 ∈ *E*_4_
*E*_5_: Edge of *G*_4_, disease *d*_*i*_ associated with LMPair *p*_*jk*_ if edge 〈*d*_*i*_, *p*_*jk*_〉 ∈ *E*_5_.

## Supplementary Materials

Supplementary Materials There are four supplementary tables in this manuscript. And among them, Supplementary Table 1 is a description of the lncRNA-disease associations, in which there are 2048 lncRNA-disease associations included, and the 1st column represents the lncRNAs and the 2nd column represents the diseases. Supplementary Table 2 is a description of the miRNA-disease associations, which contains the ID of diseases, the ID of miRNAs, and the associations between diseases and miRNAs. Supplementary Table 3 is a description of the lncRNA-miRNA associations, which contains 20343 lncRNA-miRNA associations and in which the first column represents the lncRNAs and the second column represents the miRNAs. Supplementary Table 4 is a description of the predictive results of associations between diseases and lncRNA-miRNA pairs while adopting PADLMP to execute prediction.
